# TF-GateNet: An Interpretable and Biologically Guided Framework for Primary–Metastatic State Prediction from Somatic Genomic Alterations

**DOI:** 10.3390/biom16060879

**Published:** 2026-06-15

**Authors:** Hao Zhou, Wenjia Guo, Liang He

**Affiliations:** 1 School of Computer Science and Technology, Xinjiang University, Urumqi 830017, China; zhouhao@stu.xju.edu.cn; 2 Cancer Institute, Affiliated Cancer Hospital of Xinjiang Medical University, Urumqi 830017, China; wenjiaguo@xjmu.edu.cn; 3 School of Intelligence Science and Technology, Xinjiang University, Urumqi 830017, China; 4 Xinjiang Multimodal Information Technology Engineering Research Center, Urumqi 830017, China; 5 Xinjiang Key Laboratory of Signal Detection and Processing, Urumqi 830017, China; 6 Department of Electronic Engineering, Beijing National Research Center for Information Science and Technology, Tsinghua University, Beijing 100084, China

**Keywords:** metastasis prediction, cancer genomics, biological priors, transcription factor regulation, pathway-informed modeling, interpretable deep learning, prostate cancer

## Abstract

Metastasis remains a major cause of cancer mortality, making reliable primary–metastatic state prediction from somatic genomic alterations clinically important yet technically difficult. We present TF-GateNet, a biologically constrained neural network that combines TF-aware feature integration based on TRRUST and DoRothEA TF–gene regulatory priors with sample-specific dynamic gating on a Reactome-defined hierarchical sparse backbone. The model was evaluated on multi-center prostate and breast-cancer cohorts using mutation and copy-number features across 10 repeated runs on a fixed 80/10/10 split, together with independent prostate external validation, and was compared with biologically informed neural-network baselines (P-NET, BKGNet-Pathway, and BKGNet-Protein), a dense feed-forward neural network (FNN), and conventional machine-learning baselines (LR, SVM, RF, DT, and XGBoost). On prostate, TF-GateNet achieved the best internal performance (AUROC 0.954 ± 0.005; AUPRC 0.925 ± 0.007) and the best combined external performance (AUROC 0.952 ± 0.009; AUPRC 0.898 ± 0.018). On breast, TF-GateNet achieved the strongest internal ranking performance, reaching AUROC 0.893 ± 0.004 and AUPRC 0.835 ± 0.006. Ablation analysis indicated that TF-aware integration accounted for the larger prostate gain, whereas within the TF-GateNet family on breast, both TF-aware integration and dynamic gating contributed positively. Interpretability analysis further supported a cross-level route from TF-related genomic perturbation cues to genes, pathways, and phenotype-associated predictions. These results position TF-GateNet as a biologically grounded and interpretable framework for primary–metastatic state prediction, with the strongest overall evidence in prostate cancer and favorable internal evidence in breast cancer.

## 1. Introduction

Metastatic dissemination remains a dominant cause of treatment failure and cancer mortality. Unlike many localized primary tumors that can be controlled by surgery or radiotherapy, metastatic disease is typically systemic, clinically heterogeneous, and tightly linked to adverse outcomes [1,2,3,4]. This makes early recognition of primary–metastatic state and mechanistically grounded risk stratification particularly valuable. In this study, we focus on primary–metastatic state prediction from somatic mutation and copy-number variation (CNV) profiles, with the dual aim of improving phenotype prediction and generating testable mechanistic hypotheses about metastatic programs.

High-throughput sequencing and large multi-institutional cancer registries have created an opportunity to learn metastasis-related signals from real-world genomic data. AACR Project GENIE and similar resources have aggregated clinically generated sequencing profiles across institutions, but the same diversity that increases sample size also introduces instability [5,6]. Differences in sequencing platforms, gene coverage, preprocessing pipelines, and cohort composition can produce batch effects and distribution shift, especially when mutation and CNV features are sparse and panel/WES coverage differs across centers. Consequently, models that rely only on a single static pattern shared across all samples may generalize poorly and can produce unstable explanations when applied to external cohorts. Recent benchmarking work further indicates that omics prediction models can be highly sensitive to preprocessing choices, split design, and evaluation protocol, making external validation and robustness reporting central rather than optional [7].

Deep learning has shown strong representational power in cancer diagnosis, prognosis, and treatment prediction, yet raw predictive performance alone is not sufficient in high-stakes biomedical settings [8,9]. Post-hoc attribution can provide useful clues, but it does not guarantee that the inferred explanation faithfully reflects the decision process, and explanation itself may shift when the data distribution changes [10,11,12,13]. These concerns have motivated a growing preference for intrinsically interpretable architectures in genomics, where prior biological structure is used to constrain computation and to make interpretation part of the model design rather than a retrospective add-on. Recent work on biology-inspired and interpretable deep learning likewise shows that structural readability alone does not guarantee robust or faithful explanation, and that explanation quality itself should be evaluated rather than assumed [11,14].

Biologically informed neural networks have partially addressed this need by aligning hidden layers with genes, pathways, or cellular subsystems. PASNet and Cox-PASNet introduced pathway-constrained sparse architectures; DCell and DrugCell embedded hierarchical biological systems into visible networks; P-NET demonstrated that a Reactome-based hierarchical sparse backbone can support clinically relevant cancer-state prediction with interpretable pathway-level signals; and BKGNet expanded the knowledge scope by leveraging biological knowledge graphs and adjustable connections [15,16,17,18,19,20]. The visible-network literature has also expanded beyond these representative examples to include framework-level, ontology-aware, parsimonious, and mutation-aware variants such as GenNet, Deep GONet, ParsVNN, and MPVNN, highlighting that biological priors can enter predictive models through multiple architectural loci rather than through a single pathway-mask design [21,22,23,24,25]. Outside visible sparse backbones, graph-based and attention-based methods have provided complementary strategies for cancer genomics. Graph convolutional networks, graph-attention networks, and multi-omics graph neural frameworks such as MOGONET encode molecular relations through message-passing [26,27,28], while graph-attention frameworks such as CGMega further demonstrate how attention layers can be used to dissect cancer gene modules [29]. Transformer-based omics models offer another route for learning broad feature interactions, as illustrated by biological-pathway-based transformer models for tracing unknown tumor origins [30]. These approaches highlight the value of flexible relation learning, but attention or message-passing representations are not automatically aligned with a fixed regulatory-to-pathway hierarchy. In a related cancer-state prediction setting, BKGNet also reported that an attention-network comparison was weaker than the knowledge-graph-guided design, providing a cautionary example that attention-style relation learning does not necessarily recover biologically appropriate asymmetric or global dependencies [20]. TF-GateNet therefore follows a different design philosophy: it keeps the Reactome-defined topology visible, injects TF–gene prior information before pathway propagation, and uses node-wise gates for sample-specific modulation rather than treating latent attention weights as the main interpretability route. Even so, two limitations remain central for primary–metastatic state prediction. First, many pathway-centric architectures do not provide an explicit regulatory entry from upstream TF–gene relationships. Second, fixed or globally learned connections are not ideally suited to represent sample-specific pathway utilization under marked metastatic heterogeneity.

**Figure 1:**
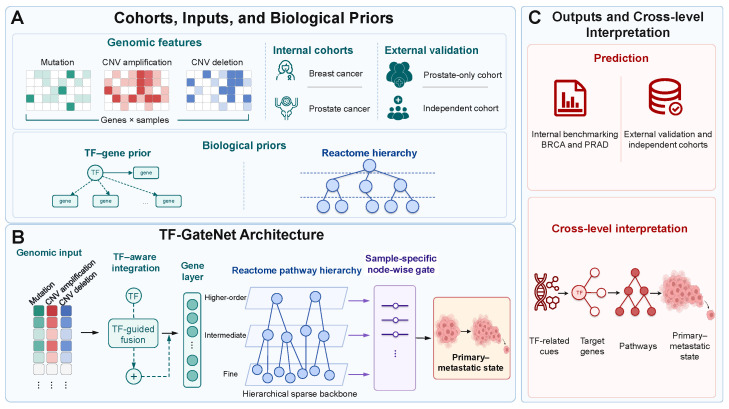
Study overview of TF-GateNet. (**A**) Cohorts, genomic features, and biological priors, including the curated TF–gene prior and the Reactome hierarchy. (**B**) TF-GateNet architecture, showing TF-guided fusion, the gene layer, the hierarchical sparse backbone, and sample-specific node-wise gating. (**C**) Outputs and cross-level interpretation, linking TF-related cues to target genes, pathways, and primary–metastatic state. This overview figure summarizes the study-level workflow, whereas the implementation-level architecture of TF-GateNet, including TF-aware feature fusion, Reactome-constrained sparse propagation, dynamic gating, and layer-wise supervision, is presented in Figure 2.

TF-GateNet was developed to address these two limitations. The framework retains a Reactome-defined hierarchical sparse backbone, while introducing TF-aware feature integration to inject TF–gene regulatory prior-guided signals into gene representations through curated resources such as TRRUST and DoRothEA [31,32]. Because the inputs are genomic alterations rather than direct assays of TF activity, these signals are interpreted here as TF-related genomic perturbation cues rather than direct measurements of TF activity. The model further incorporates sample-specific dynamic gating, implemented as node-wise gating across pathway layers, so that pathway-node activations can vary from sample to sample without breaking the prior-defined topology. Figure 1 summarizes the overall design. Panel A links genomic features, internal cohorts, external validation, and biological priors; Panel B outlines TF-guided fusion, the gene layer, the Reactome pathway hierarchy, and the node-wise gate; and Panel C connects prediction outputs to a cross-level interpretation route from TF-related cues to target genes, pathways, and primary–metastatic state. The study therefore proceeds along three complementary lines of evidence: internal benchmarking on breast and prostate cohorts, external validation on independent prostate cohorts, and cross-level interpretation from TF-related perturbation cues to phenotype-associated predictions. Taken together, these analyses contribute a regulatory prior-guided entry, dynamic pathway modulation, and an interpretation route spanning genes, pathways, and phenotype-associated prediction.

**Figure 2:**
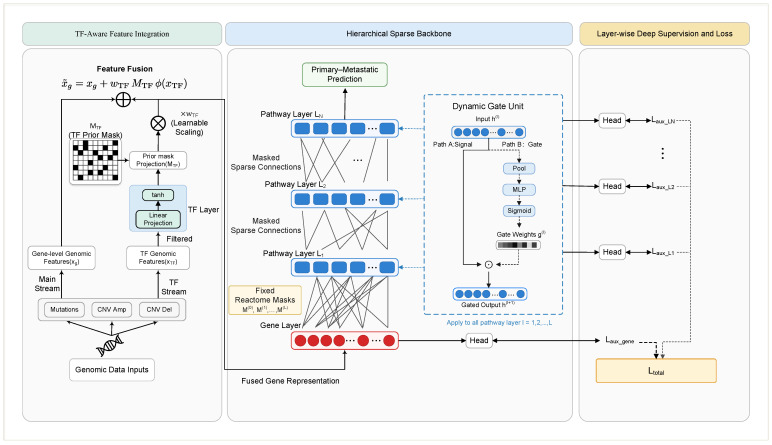
Detailed architecture of TF-GateNet. TF-GateNet receives three somatic genomic-alteration channels, including mutation, copy-number amplification, and copy-number deletion. The main stream preserves gene-level genomic features, whereas the TF-aware stream extracts TF genomic features and projects them through a binary TF–gene prior mask $M_{\mathrm{TF}}$, constructed from the union of TRRUST and DoRothEA regulatory interactions. The resulting regulatory-context signal is fused with the main gene-level representation through a learnable scaling factor $w_{\mathrm{TF}}$. The fused gene representation is then propagated through a Reactome-defined hierarchical sparse backbone, where fixed Reactome masks restrict trainable weights to prior-supported edges. A dynamic gate is applied to each pathway layer to generate sample-specific node-wise modulation weights. The gate modulates pathway-node activations but does not alter the Reactome-defined sparse topology or create new edges. Layer-wise heads provide deep supervision at the gene and pathway levels, while the model output estimates the primary–metastatic state. This architecture preserves a traceable route from TF-related genomic perturbation cues to genes, pathways, and phenotype-associated prediction.

## 2. Materials and Methods

### 2.1. Study Design and Problem Formulation

We formulated the task as a binary prediction of primary versus metastatic tumor state from somatic genomic alterations. Each sample is represented by three genomic channels—mutation, CNV amplification, and CNV deletion—and the model outputs a metastatic probability. TF-GateNet combines two central design elements within one biologically constrained architecture: TF-aware feature integration at the regulatory entry and sample-specific dynamic gating implemented as node-wise gating across pathway layers. Figure 1 provides a compact overview of the study design, model architecture, and interpretation workflow.

### 2.2. Overall Architecture of TF-GateNet

TF-GateNet was designed as a biologically constrained architecture that links somatic genomic alterations to primary–metastatic state prediction through an explicitly traceable regulatory and pathway hierarchy (Figure 2). The model receives three gene-level input channels, including somatic mutation, copy-number amplification, and copy-number deletion. These channels are organized into a main gene stream and a TF-aware regulatory stream. The main stream preserves the gene-level genomic-alteration profile, whereas the TF-aware stream extracts genomic perturbation signals from genes annotated as transcription factors and projects them through a curated TF–gene regulatory prior.

In the TF-aware stream, the regulatory prior is represented by a binary TF–gene mask $M_{\mathrm{TF}}$, constructed from the union of TRRUST and DoRothEA interactions after mapping to the analyzed gene space. This branch does not infer TF activity directly from transcriptomic or chromatin measurements; rather, it provides a regulatory-context signal derived from somatic alterations affecting TFs and their prior-linked target genes. The projected TF-related representation is fused with the main gene-level representation through a learnable scaling factor $w_{\mathrm{TF}}$, producing the fused gene representation. In this way, TF-GateNet introduces upstream regulatory prior knowledge before information enters the pathway hierarchy.

The fused gene representation is then propagated through a Reactome-defined hierarchical sparse backbone. Connections from genes to lower-level pathways and between successive pathway layers are constrained by fixed Reactome masks $M^{\left(0\right)},M^{\left(1\right)},\ldots ,M^{\left(L\right)}$, so trainable weights are learned only on prior-supported edges. This design differs from a fully connected neural network because the hidden nodes correspond to biological entities and the information flow follows curated gene–pathway and pathway–pathway relationships. The resulting topology therefore remains biologically interpretable by construction.

To accommodate sample-level heterogeneity, TF-GateNet applies a dynamic gate to each pathway layer. For a pathway layer *l*, the gate generates a node-wise modulation vector $g^{\left(l\right)}$ from the layer-specific representation and applies element-wise modulation to the pathway-node activations. Importantly, this gate modulates the activation strength of existing pathway nodes in a sample-specific manner; it does not alter the Reactome-defined sparse topology, create new edges, or remove prior-defined edges. The gate should therefore be interpreted as sample-specific pathway-node activation modulation rather than topology relearning.

Layer-wise heads are attached to the gene layer and pathway layers to provide deep supervision during training, and their outputs are used within the model readout for primary–metastatic state estimation. Because TF features, gene nodes, pathway nodes, gate values, and prediction heads remain aligned with biological entities, the architecture preserves a cross-level interpretation route from TF-related genomic perturbation cues to genes, pathways, and phenotype-associated prediction.

### 2.3. Cohorts and Preprocessing

Internal evaluation was performed on prostate and breast-cancer cohorts with matched somatic mutation and CNV data. The internal prostate cohort followed the curated prostate dataset used in the P-NET study [19]. The internal breast cohort was assembled from four cBioPortal studies—BRCA IGR 2015, the 2017 Metastatic Breast-Cancer Project release, the 2022 Metastatic Breast-Cancer Project release, and the TCGA PanCancer Atlas 2018 Breast Invasive Carcinoma study—with matched somatic mutation and copy-number profiles [33]. The final aligned breast analysis set contained 1572 samples (1111 primary and 461 metastatic), with 4316 genes in the mutation channel and 19,041 genes in the CNV channel. The final aligned prostate analysis set used for model fitting contained 1011 samples (678 primary and 333 metastatic), with 14,378 genes in the mutation channel and 13,802 genes in the CNV channel. The analyzed set corresponds to the feature-aligned cohort used in the repeated-seed experiments summarized in Table 1.

The analysis was intentionally restricted to somatic mutation and CNV features because these alteration types were available in a matched form across the included public cohorts and directly reflected the somatic genomic-alteration setting targeted by the study. Transcriptomic and methylation profiles were not included because they were not uniformly available with primary–metastatic labels and matched mutation/CNV profiles across the same cohort sources. Including such modalities would have introduced substantial modality-specific missingness and platform heterogeneity, making it difficult to separate architectural effects from data-availability effects. We therefore treated multimodal molecular integration as an important extension rather than as part of the present mutation/CNV-based benchmark.

Clinical records were used to derive binary labels (primary = 0, metastatic = 1). Mutation tables were collapsed to gene-level mutation-count features after important-only filtering of non-informative event types, whereas CNV profiles were decomposed into separate amplification and deletion channels.

CNV amplification and deletion were kept as separate channels because copy-number gains and losses often have different biological implications, for example, through oncogene activation and tumor-suppressor loss. Collapsing them into a single CNV-abnormality channel would remove this directionality and could obscure asymmetric gain- and loss-associated signals. The three-channel encoding therefore preserved mutation-count features, amplification, and deletion as distinct but gene-aligned genomic-alteration patterns.

Samples were aligned across label, mutation, and CNV tables by sample identifier, and the retained analysis set was defined by the intersection of samples with available clinical labels, mutation profiles, and CNV profiles. During breast cohort assembly, sample identifiers and available patient identifiers were checked across the included cBioPortal studies. Overlap was detected between the two Metastatic Breast-Cancer Project releases and was handled by retaining the first occurrence under the predefined study order before final sample matching. Features were then harmonized to a common gene space so that internal testing and external validation shared the same input dimension.

For the breast cohort, study-level variability was handled through a uniform preprocessing and harmonization workflow rather than by fitting study-specific models. Across the four cBioPortal breast studies, primary–metastatic labels were derived using consistent label rules, mutation profiles were represented as gene-level mutation-count features after important-only filtering, and CNV profiles were represented as separate amplification and deletion channels. Only samples with matched clinical labels, mutation profiles, and CNV profiles were retained, and features were aligned to a shared gene space before model fitting. Sample and available patient identifiers were checked across studies, and overlap between the two Metastatic Breast-Cancer Project releases was handled by duplicate removal before final sample matching. These steps reduced differences caused by study-specific sample availability and feature coverage, although residual differences in study design, feature availability after common-space harmonization, mutation/CNV processing, sample ascertainment, treatment history, clinical annotation, and cohort composition could not be fully removed and are addressed as limitations in the Discussion. At the time of analysis, no independent public breast cohort satisfying the same primary–metastatic label, matched mutation/CNV, and compatible feature-space requirements was available from the surveyed cBioPortal resources; therefore, independent external validation was performed only for prostate cancer.

External prostate validation used two independent cBioPortal studies: a primary prostate cohort from TCGA PanCancer Atlas and a metastatic cohort from the SU2C 2015 prostate study [33]. These cohorts were processed with the same feature construction and alignment pipeline as the internal data, and no retraining or additional hyperparameter tuning was performed on the external cohorts.

### 2.4. Hierarchical Sparse Backbone

To enforce biological structure, we constructed a hierarchical sparse backbone from the Reactome pathway hierarchy [34]. Aligned input genes were first mapped to a gene layer and then recursively connected to multiple pathway layers through child–parent relations. Not all genes from the raw mutation/CNV input space were retained in the final backbone: only genes that could be mapped to Reactome and preserved effective support in the analyzed cohort were kept. For prostate, the resulting backbone contained 7 total layers (1 gene layer and 6 pathway layers), with 9229, 1610, 1116, 502, 167, 29, and 1 nodes from the gene layer to pathway layer 6, respectively. The corresponding edge counts were 18,351 gene-to-pathway edges and 3439 pathway-to-pathway edges, for 21,790 total effective edges. For breast, the backbone also contained 7 total layers, with 10,267, 1610, 1116, 502, 167, 29, and 1 nodes across the same hierarchy, connected by 25,779 gene-to-pathway edges and 3439 pathway-to-pathway edges, yielding 29,218 total effective edges.

The six pathway layers were not selected as a tuned hyperparameter. They corresponded to the retained Reactome child–parent hierarchy after mapping the analyzed genes to Reactome and pruning unsupported nodes. Thus, both cancer-specific backbones used one gene layer followed by the six preserved Reactome pathway levels, with cancer-specific differences arising from the genes retained after feature alignment and Reactome mapping.

Table 2 summarizes the cancer-specific input-space and TF regulatory-entry statistics used in TF-GateNet.

For each pair of adjacent layers, only prior-supported edges were retained by a binary mask $M^{\left(l\right)}$. Given hidden representation $h^{\left(l\right)}$, the masked transform was defined as

(1) $$h^{\left(l+1\right)}=f\;\left({\left(M^{\left(l\right)}⊙W^{\left(l\right)}\right)}^{⊤}h^{\left(l\right)}+b^{\left(l\right)}\right),$$

where f(·) denotes the activation function. Lightweight readout heads were attached to the gene layer and each pathway layer, and the final metastatic probability was obtained by averaging the readout probabilities across levels.

### 2.5. TF-Aware Feature Integration

Before information enters the hierarchical sparse backbone, TF-GateNet injects a regulatory prior at the gene level. We first identified the subset of TF genes available in the aligned genomic input space and then merged curated TF–gene interactions from DoRothEA and TRRUST by union to form the regulatory prior mask $M_{\mathrm{TF}}$ [31,32]. At the source level, DoRothEA contributed 13,223 usable edges spanning 5388 nodes, whereas TRRUST contributed 8427 usable edges spanning 2862 nodes. Their union contained 18,495 edges over 6284 nodes. After intersection with the cancer-specific analyzed gene spaces, the final prostate model retained 448 effective TFs and 6769 effective TF–gene edges, while the breast model retained 685 effective TFs and 13,757 effective edges. This module does not attempt to estimate TF activity directly. Instead, it injects TF-related genomic perturbation cues into gene representations through curated regulatory priors.

Confidence annotations from the source resources were not used as continuous edge weights in the present model. Because DoRothEA and TRRUST provide regulatory evidence in different formats and the available interactions must be mapped to cancer-specific genomic feature spaces, we used a binary union mask after gene-space intersection to prioritize coverage and cross-cohort compatibility. Confidence-weighted TF–gene edges may provide a useful extension, but they would require additional calibration across resources and cohorts.

Let $x_{g}$ denote the gene-level input and $x_{\mathrm{TF}}$ the TF subset. TF-aware feature integration is defined as

(2) $${\tilde{x}}_{g}=x_{g}+w_{\mathrm{TF}}\;M_{\mathrm{TF}}\;\phi \left(x_{\mathrm{TF}}\right),$$

where ϕ(·) is a learnable nonlinear transform and $w_{\mathrm{TF}}$ is a scalar balancing coefficient.

### 2.6. Sample-Specific Dynamic Gating

To capture individualized pathway utilization on a fixed topology, we apply sample-specific dynamic gating to every pathway layer. For layer *l*, the masked linear output is

(3) $$y^{\left(l\right)}={\left(M^{\left(l\right)}⊙W^{\left(l\right)}\right)}^{⊤}h^{\left(l\right)}+b^{\left(l\right)},$$

a gate input is built by prior-guided neighborhood aggregation,

(4) $$a^{\left(l\right)}=\mathrm{Norm}\;\left({\left(M^{\left(l\right)}\right)}^{⊤}h^{\left(l\right)}\right),$$

followed by temperature-controlled gating

(5) $$g^{\left(l\right)}=\sigma \;\left((s^{\left(l\right)}⊙a^{\left(l\right)}+c^{\left(l\right)})/T\right),$$

and node-wise modulation

(6) $$h^{\left(l+1\right)}=f\;\left(g^{\left(l\right)}⊙y^{\left(l\right)}\right).$$

Here, $g^{\left(l\right)}$ can be interpreted as sample-specific pathway participation strength at layer *l*. Smaller temperatures encourage sharper selection, whereas larger temperatures yield smoother amplitude modulation.

### 2.7. Loss Function, Baselines, and Training Protocol

The training objective combines classification supervision with gate regularization. Let ${\hat{y}}^{\left(m\right)}$ denote the output of readout head *m*. The classification loss is a weighted multi-head binary cross-entropy,

(7) $$L_{\mathrm{cls}}=\sum_{m=1}^{M}\alpha_{m}\;\mathrm{BCE}\;\left(y,{\hat{y}}^{\left(m\right)}\right).$$

For each gated layer, we add an L1 term to encourage selective pathway use and an entropy term to avoid premature saturation:

(8) $$L_{\mathrm{gate}}=\sum_{l\in G}\left[\lambda_{1}\;\mathrm{mean}\;\left(g^{\left(l\right)}\right)+\lambda_{H}\;\mathrm{mean}\;\left(-g^{\left(l\right)}\log g^{\left(l\right)}-\left(1-g^{\left(l\right)}\right)\log\left(1-g^{\left(l\right)}\right)\right)\right].$$

The final objective is $L=L_{\mathrm{cls}}+\beta \;L_{\mathrm{gate}}$.

All internal experiments used the same fixed stratified 80/10/10 split within each cancer type, and all compared models shared the identical split. Keeping the split unchanged, each experiment was repeated across 10 random seeds and summarized as mean ± sd. Validation AUPRC was used as the primary criterion for model selection, checkpoint selection, and learning-rate scheduling. Given the class imbalance of the cohorts, AUROC and AUPRC were treated as the primary ranking metrics throughout model selection and result interpretation, with particular emphasis on AUPRC in line with methodological guidance for imbalanced binary classification [35,36].

The baselines included LR, linear SVM, RF, DT, XGBoost, FNN, P-NET, BKGNet-Pathway, and BKGNet-Protein. For baseline configuration, biology-informed deep-learning baselines were implemented using the architectural and training hyperparameters reported in their corresponding papers or released implementations whenever available, whereas classical machine-learning baselines were evaluated with default estimator hyperparameters, with only the random seed varied where applicable. The dense FNN baseline was implemented as a topology-relaxed counterpart of the retained Reactome hierarchy. It preserved the same cancer-specific layer depth and layer widths as the corresponding Reactome backbone described in Section 2.4, but all prior-supported sparse masks between adjacent layers were removed and replaced with fully connected transformations. Specifically, the dense FNN used six hidden transformations with layer widths inherited from the cancer-specific backbone: 9229, 1610, 1116, 502, 167, 29, and 1 nodes for prostate, and 10,267, 1610, 1116, 502, 167, 29, and 1 nodes for breast, from the gene layer to the top pathway layer. The FNN did not use the TF-aware regulatory branch or sample-specific dynamic gates. Hidden layers used tanh activation with layer-wise dropout rates of 0.5, 0.1, 0.1, 0.1, 0.1, 0.1, and 0.1. The dense FNN was trained with Adam, an initial learning rate of $10^{-3}$, weight decay of $10^{-3}$, batch size 50, a maximum of 250 epochs, automatic class weighting, and early stopping enabled with patience 40. Gate-specific regularization terms were not applied. This design allowed the FNN to serve as a dense neural-network baseline for separating the effect of biological sparse topology from the shared hierarchical layer-width design. All deep-learning models, including TF-GateNet and the neural-network baselines, used the same validation-AUPRC-based checkpoint selection and early-stopping strategy. Threshold-based metrics, including F1, ACC, MCC, BACC, and cohort-specific correct rates, were computed using a fixed metastatic-probability threshold of 0.5.

TF-GateNet was optimized with Adam using a batch size of 32, a maximum of 250 epochs, an initial learning rate of $10^{-3}$, and weight decay of $10^{-3}$. Minority-class weighting was applied in the binary cross-entropy loss to compensate for label imbalance. The learning rate was halved when validation AUPRC failed to improve for 10 epochs, with a floor of $10^{-6}$. Hierarchical readout weights were fixed to [2,7,20,54,148,400]. In the final frozen configuration, dynamic gating was applied to all 6 pathway layers (pathway_1_–pathway_6_) rather than to the TF entry branch, and the gate-specific hyperparameters were fixed to temperature T=0.15, L1 regularization coefficient $\lambda_{1}=0.2$, and entropy regularization coefficient $\lambda_{H}=0.2$. The final prediction was obtained by averaging the readout heads, and all experiments were run on an NVIDIA GeForce RTX 4090 under Ubuntu 20.04.

Ablation experiments were retrained from scratch under the same split and seed protocol and were analyzed within their own comparison workflow. Pairwise statistical comparisons were performed on seed-wise AUPRC values, because AUPRC was the primary metric for imbalanced primary–metastatic prediction. For each cancer cohort and comparison family, each baseline or ablation variant was compared with TF-GateNet using a paired Wilcoxon signed-rank test across the same 10 random seeds. The pairing was defined by the random seed so that each comparison used matched runs generated under the same fixed train/validation/test split. Multiple comparisons were adjusted using the Benjamini–Hochberg false-discovery-rate procedure within each cancer-specific AUPRC comparison family. The resulting adjusted values are reported as *q*(AUPRC).

## 3. Results

### 3.1. Internal Prediction Performance on Prostate and Breast Cohorts

**Figure 3:**
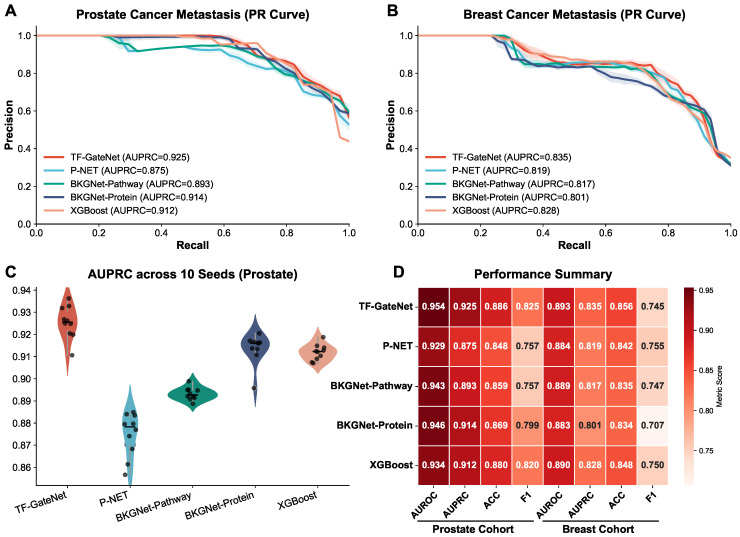
Internal benchmarking across the prostate and breast cohorts. (**A**,**B**) Seed-averaged precision–recall curves for TF-GateNet, P-NET, BKGNet-Pathway, BKGNet-Protein, and XGBoost on the prostate and breast test sets; legends report mean AUPRC across 10 runs and shaded bands denote seed-level variability. (**C**) Seed-wise AUPRC distributions for representative models on the prostate cohort. (**D**) Heatmap summarizing mean AUROC, AUPRC, ACC, and F1 across both cohorts.

Table 3 summarizes the internal test performance on the two benchmark cohorts across 10 repeated runs on the fixed 80/10/10 split. Because the class distribution is imbalanced and the preferred operating threshold may vary across applications, AUROC and AUPRC were treated as the primary evaluation metrics, whereas F1, ACC, and MCC were used to assess threshold-based classification behavior at a fixed metastatic-probability threshold of 0.5. Figure 3 provides the main visual summary of these results. This section establishes the first line of evidence for TF-GateNet, namely strong internal ranking performance on both cancers, with the clearest overall support observed in prostate cancer.

On the prostate cohort, TF-GateNet achieved the strongest overall performance among all compared models, reaching AUROC 0.954 ± 0.005 and AUPRC 0.925 ± 0.007. Relative to P-NET, this corresponded to gains of 0.025 in AUROC and 0.050 in AUPRC; relative to BKGNet-Protein, the gains were 0.008 and 0.011, respectively. The AUPRC improvement over P-NET, both BKGNet variants, and each empirical baseline remained significant after multiple-testing correction, indicating that the TF-aware regulatory entry and gated hierarchical backbone added predictive information beyond pathway-only and purely empirical alternatives. Threshold-based metrics were aligned with the ranking metrics on prostate: TF-GateNet also achieved the highest mean F1, ACC, and MCC. To keep the main figure compact, Figure 3 shows the prostate and breast PR curves, the seed-wise prostate AUPRC distributions for representative models, and a heatmap summary of the mean metrics across both cohorts.

The breast cohort also showed strong internal performance for TF-GateNet. The model achieved the best mean AUROC (0.893 ± 0.004) and the best mean AUPRC (0.835 ± 0.006) among all compared methods. It exceeded P-NET, BKGNet-Pathway, BKGNet-Protein, and XGBoost by 0.016, 0.018, 0.034, and 0.007 AUPRC points, respectively. In addition to its ranking advantage, TF-GateNet also achieved the highest mean ACC, whereas XGBoost showed a marginally higher F1 and TF-GateNet retained the higher MCC. Therefore, the breast benchmark is better interpreted as favorable internal evidence for TF-GateNet rather than as a merely mixed or near-parity pattern. Taken together, the internal benchmarking supports TF-GateNet on both cancers, while also establishing the prostate cohort as the setting with the strongest overall evidence. Complete seed-wise internal benchmarking metrics and pairwise statistical comparisons are provided in Supplementary Tables S1 and S2.

### 3.2. Ablation of TF-Aware Integration and Dynamic Gating

**Figure 4:**
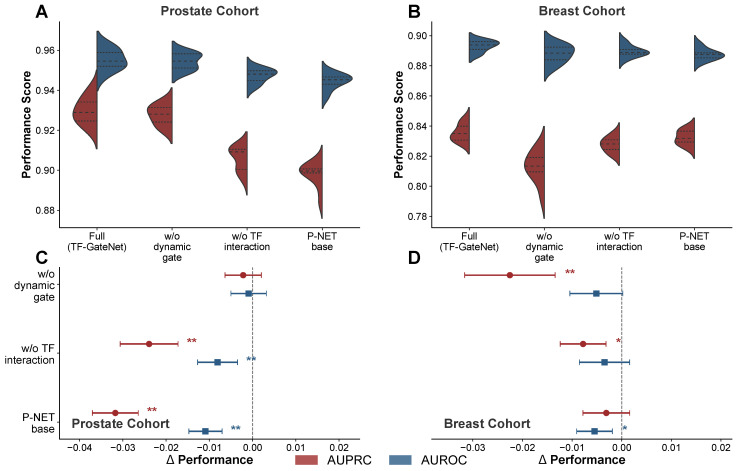
Ablation summary across the prostate and breast cohorts. (**A**,**B**) Seed-wise AUPRC distributions for the four ablation variants used in the module-wise comparisons: Full TF-GateNet, w/o Gate (P-NET+TF), w/o TF (P-NET+Gate), and P-NET. (**C**) Effect-size summary showing ΔAUPRC (Full-comparator) for w/o TF, w/o Gate, and P-NET on prostate and breast. (**D**) Heatmap of Benjamini–Hochberg-adjusted AUPRC q-values from paired seed-wise comparisons against the full TF-GateNet model. Asterisks indicate Benjamini–Hochberg-adjusted AUPRC significance against the full TF-GateNet model in paired seed-wise comparisons (* q < 0.05; ** q < 0.01).

We next examined how the two additional components of TF-GateNet shaped the internal performance pattern described above. Table 4 focuses on the effect of TF-aware feature integration, whereas Table 5 isolates the contribution of dynamic gating. Figure 4 summarizes the same comparisons visually through seed-wise distributions, effect-size contrasts, and q-value heatmaps. Because these ablation experiments were retrained from scratch within a dedicated comparison workflow, the full TF-GateNet entries in the ablation tables are not expected to be numerically identical to those in the main benchmarking table. The most informative quantities are therefore the within-workflow differences relative to the full TF-GateNet model, rather than exact numerical identity with Table 3.

On the prostate cohort, the ablation results consistently pointed to a stronger TF-driven contribution. Adding TF-aware feature integration increased AUPRC in both the P-NET family and the TF-GateNet family, and the corresponding gains were larger than those produced by dynamic gating alone. Dynamic gating was still beneficial, but its in-cohort effect was more modest. In other words, the larger internal gain on the prostate was primarily associated with the TF-aware regulatory entry.

In the breast cohort, the pattern was different and depended more strongly on architectural context. In the P-NET family, adding TF-aware integration or dynamic gating did not uniformly improve AUPRC. By contrast, within the TF-GateNet family, both modules were beneficial, and the larger drop appeared after removing dynamic gating. The key message on breast is therefore not that either module acts as a universal plug-in improvement across all family settings, but that the coordinated full TF-GateNet architecture produced the strongest internal ranking performance.

Taken together, the ablation analysis explains the internal benchmark in mechanistic terms. TF-aware feature integration accounted for the larger prostate gain, whereas on breast the favorable internal result depended more strongly on the coordinated full architecture, with dynamic gating contributing more clearly within the TF-GateNet family. Complete seed-wise ablation metrics and pairwise statistical comparisons are provided in Supplementary Tables S3 and S4.

### 3.3. External Prostate Validation

**Figure 5:**
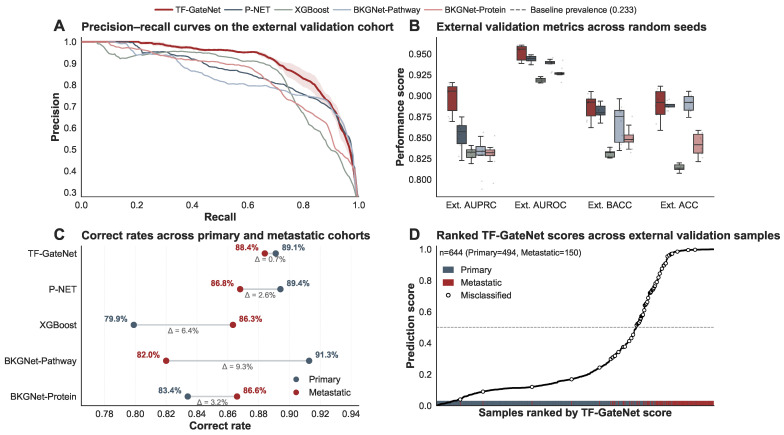
External validation on independent prostate cohorts from cBioPortal. (**A**) Precision–recall curves for TF-GateNet, P-NET, BKGNet-Pathway, BKGNet-Protein, and XGBoost; the gray dashed reference marks the metastatic-class prevalence (150/644 = 0.233). (**B**) External-validation metrics across 10 runs for AUPRC, AUROC, BACC, and ACC, with threshold-based metrics computed at a fixed metastatic-probability threshold of 0.5. (**C**) Correct rates across the primary and metastatic external cohorts, with the paired-point layout highlighting cohort balance and the within-model gap Δ. (**D**) Ranked TF-GateNet scores across external-validation samples (n=644; 494 primary and 150 metastatic), where the black curve indicates the ranked prediction score, the bottom rug indicates the true cohort label and hollow circles denote misclassified samples.

To test whether the gains observed internally could be maintained under cohort shift, we evaluated the internally selected models on an independent prostate external validation setting composed of a primary cohort from the TCGA PanCancer Atlas and a metastatic cohort from the SU2C 2015 prostate study. No retraining or hyperparameter retuning was performed on the external data. Table 6 summarizes the numerical external-validation results, whereas Figure 5 provides a complementary visual overview of ranking performance, seed-wise stability, cohort balance, and sample-level score behavior on the independent prostate cohorts.

As summarized in Table 6 and visualized in Figure 5, TF-GateNet retained the best combined external ranking performance, reaching an external AUROC of 0.952 ± 0.009 and external AUPRC of 0.898 ± 0.018. Because the metastatic-class prevalence in the external validation set was 0.233 (150/644), the observed AUPRC was far above the random-ranking reference level expected under this class imbalance. Its external AUPRC exceeded that of P-NET, BKGNet-Pathway, BKGNet-Protein, and XGBoost by 0.046, 0.068, 0.068, and 0.067, respectively, and it also achieved a near-best external ACC (0.889 ± 0.018) and the highest BACC (0.888 ± 0.015). The external AUPRC differences versus P-NET, BKGNet-Pathway, FNN, and XGBoost remained significant after multiple-testing correction, and BKGNet-Protein was also clearly lower than TF-GateNet.

The advantage was not driven by only one side of the external split. TF-GateNet achieved a primary-cohort correct rate of 0.891 ± 0.026 and a metastatic-cohort correct rate of 0.884 ± 0.026, yielding the most balanced behavior across the two external sources. Some comparison models scored higher on one side—for example, FNN and BKGNet-Pathway on the primary cohort—but they dropped more markedly on the opposite side or on the combined balance metrics. This indicates that TF-GateNet’s advantage lies not only in overall ranking but also in more stable cross-source behavior.

Together with the ablation findings, the external validation clarifies the role of dynamic gating. Although dynamic gating produced only a modest internal AUPRC gain on prostate, the full model achieved the strongest external ranking and the best source balance. This suggests that dynamic gating may be more relevant for portability under cohort shift than for maximizing a single in-cohort summary score. This emphasis on independent-cohort testing is consistent with recent benchmarking guidance for omics prediction models, which increasingly treats external validation as a core criterion rather than an optional add-on [7]. Complete seed-wise external validation metrics and pairwise statistical comparisons are provided in Supplementary Tables S5 and S6.

### 3.4. Interpretability Analysis

Beyond internal ranking performance and external portability, we asked whether TF-GateNet also preserved a biologically traceable decision route. Because the model was designed as an intrinsically interpretable architecture rather than a black box followed by post hoc explanation, the interpretability analysis focused on three complementary questions: how pathway gating changed with predicted risk, how strongly dominant pathways recurred across samples, and whether cross-level attribution remained anchored to observable genomic abnormalities. In this sense, the interpretability pipeline traces evidence from TF-related genomic perturbation cues to genes, pathways, and phenotype-associated predictions, providing the third line of evidence for TF-GateNet.

It is also important that this analysis was carried out on the full cancer-specific architecture rather than on a simplified toy network. In the prostate setting, the model included 448 effective TFs and 6769 effective TF–gene edges within a sparse hierarchy containing 9229 gene-layer nodes and six pathway layers. These structural scales define the actual interpretability space over which gating and attribution were evaluated.

**Figure 6:**
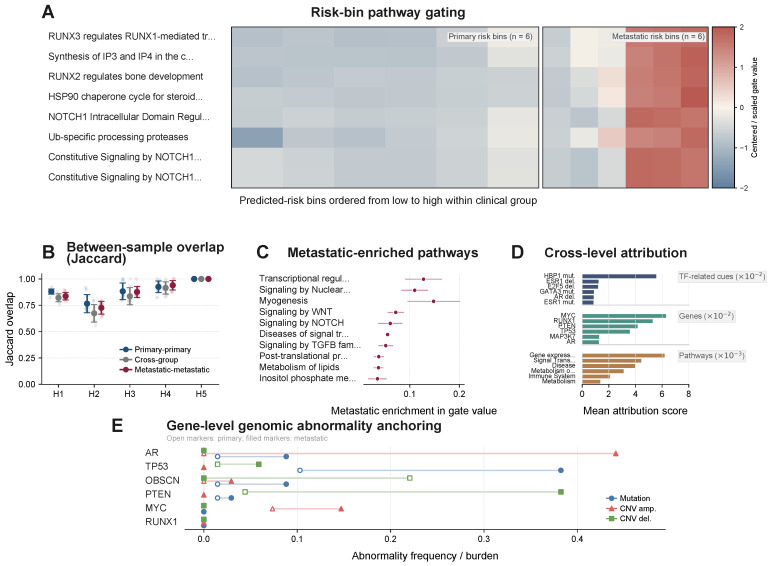
Interpretability analysis of TF-GateNet on the prostate cohort. (**A**) Risk-bin pathway gating across predicted-risk bins within the primary and metastatic groups. (**B**) Between-sample overlap of dominant pathways, summarized by Jaccard similarity for primary–primary, cross-group, and metastatic–metastatic comparisons. (**C**) Pathways showing higher gate values in metastatic samples. (**D**) Cross-level attribution across TF-related genomic perturbation cues, genes, and pathways. (**E**) Gene-level genomic abnormality anchoring for recurrently prioritized genes; open markers denote primary samples and filled markers denote metastatic samples. Truncated labels in panels A, C, and D are used for visual readability and do not affect scientific interpretation; full ranked interpretability outputs are provided in Supplementary Table S7.

Figure 6 summarizes the results from five complementary views. Figure 6A shows that pathway usage varied not only between primary and metastatic predictions but also across ordered risk bins within each clinical group. Figure 6B quantifies overlap among dominant pathways, indicating a mixture of partial recurrence and sample-specific modulation. Figure 6C highlights pathways with higher gate values in metastatic samples, including recurrent enrichment of signaling and metabolic programs such as inositol phosphate metabolism, lipid metabolism, TGFβ-related signaling, NOTCH, WNT, and nuclear receptor-associated pathways. Figure 6D follows attribution across TF-related perturbation cues, genes, and pathways, linking upstream genomic abnormalities to downstream phenotype-associated importance. Figure 6E anchors recurrently prioritized genes to observed mutation and CNV burden, showing that highly weighted nodes remained connected to measurable genomic abnormalities rather than emerging as purely mathematical artifacts.

Taken together, these analyses suggest that TF-GateNet does not rely on a single isolated marker. Instead, metastasis-associated behavior is captured as a structured multi-level signal, reflected in coordinated pathway use, partial cross-sample recurrence, cross-level attribution consistency, and genomic abnormality anchoring of prioritized genes. The interpretability results therefore complete the study narrative by supporting a coherent evidence route from TF-related genomic perturbation cues to genes, pathways, and primary–metastatic state prediction. Full ranked interpretability outputs, including TF-related cues, genes, pathways, and risk-bin pathway summaries, are provided in Supplementary Table S7.

To assess whether the prioritized interpretability signals were driven by a single training realization, we further summarized seed-wise recurrence of top-ranked TF-related cues, genes, pathways, gate-derived pathway signals, and cross-level attribution edges across the 10 repeated runs. Supplementary Table S9 reports mean pairwise top-k Jaccard overlap across the 45 seed pairs for k = 10, 20, and 50, together with recurrent top-20 interpretability signals. The stability summary showed partial but consistent recurrence of prioritized signals, with moderate recurrence for TF-related cue and gene attribution, layer-dependent recurrence across pathway-attribution summaries, moderate top-10 and top-20 recurrence for metastatic-enriched gate pathways, and lower but non-zero recurrence for cross-level attribution edges. These results support the robustness of candidate interpretability signals across repeated training seeds, while the biological interpretation remains hypothesis-generating rather than causal.

## 4. Discussion

This study was designed to improve primary–metastatic state prediction without giving up biological traceability, and the current results support a coherent three-part narrative. The first line of evidence comes from internal benchmarking, where TF-GateNet showed the strongest overall support on prostate and favorable internal evidence on breast. The second comes from the combined ablation and external-validation analyses. These results suggest that TF-aware feature integration accounted for the larger internal gain on prostate, whereas dynamic gating contributed more clearly to portability under cohort shift and to the coordinated full-architecture benefit observed on breast. The third comes from interpretability analysis, where pathway gating, cross-level attribution, and genomic abnormality anchoring together supported a biologically traceable route from TF-related perturbation cues to phenotype-associated prediction. Taken together, these findings position TF-GateNet as a biologically informed framework that combines a regulatory prior-guided entry with dynamic pathway modulation for clinically relevant metastasis prediction. Viewed against this broader literature, TF-GateNet is best understood as part of a recent shift from fixed visible backbones toward biologically constrained models that retain structural interpretability while allowing more adaptive internal computation [25,37,38,39].

TF-GateNet should therefore not be viewed as preferable solely through the lens of whether it achieves the highest numerical score in every metric or cohort. Its main advantage lies in combining competitive predictive performance with a prior-constrained and inspectable architecture: a TF regulatory entry, a fixed Reactome sparse topology, sample-specific node-wise modulation, and cross-level traceability from TF-related genomic perturbation cues to phenotype-associated prediction. This distinction is important because, in biomedical prediction, a modest or cohort-dependent performance gain can still be meaningful when the architecture also constrains the hypothesis space and makes the route from genomic alteration to prediction more auditable.

The different prostate and breast patterns should also be interpreted in this architectural context. In prostate cancer, TF-aware integration produced the larger internal gain, and the full model retained strong external performance, consistent with a good match between the regulatory entry, the prostate data structure, and the Reactome-constrained hierarchy used here. In breast cancer, TF-GateNet achieved the strongest internal ranking performance, but the absolute margins over the closest comparators were smaller, and no independent breast external validation was available. The breast results are therefore best interpreted as favorable internal evidence for the coordinated TF-GateNet architecture rather than as definitive evidence of broad breast-cancer generalization. The more modest separation may partly reflect the multi-source WES/WXS cohort composition of the breast analysis, source-specific primary/metastatic imbalance, differences in feature availability after common-space harmonization, clinical annotation, treatment-history availability, and the biological diversity of breast-cancer subtypes, although these explanations remain hypotheses rather than demonstrated mechanisms.

Several limitations should be acknowledged. First, the model depends on the coverage and quality of curated priors, and both Reactome and TF–gene resources may contain incomplete or context-mismatched relations. More generally, recent methodological work increasingly treats biological priors as confidence-bearing and context-dependent scaffolds rather than as uniformly reliable ground truth, which is directly relevant to how TF-related and pathway-level findings should be interpreted here [7,25]. Second, the TF input branch should be interpreted cautiously: mutation and CNV patterns in TF genes are TF-related genomic perturbation cues rather than direct measurements of TF activity. Third, the current study focuses on mutation and CNV only, and broader molecular layers may improve both prediction and mechanistic resolution. Finally, only prostate received external validation in the current version, so broader multi-cancer validation remains necessary.

Dataset scale and cohort composition further constrain how broadly the results can be interpreted. Although the internal cohorts contained more than one thousand samples in each cancer type, the effective evaluation of metastatic prediction depends on the smaller metastatic subsets and on the fixed train/validation/test partitions. These data are informative for method development and repeated-seed benchmarking, but they are not designed to fully resolve subtype-specific effects, stable rare-event patterns, or platform-specific generalization. This limitation is most relevant for the breast analysis. At the time of analysis, the public cBioPortal breast datasets that met the primary–metastatic label and matched mutation/CNV requirements were drawn from multiple WES/WXS-based sources rather than from a single uniformly ascertained cohort. The assembled breast cohort combined metastatic-enriched sources, two Metastatic Breast-Cancer Project releases, and a primary-dominant TCGA source, with overlap between the two Metastatic Breast-Cancer Project releases handled by duplicate removal before final sample matching. This multi-source design increased sample availability but may introduce source-composition bias and label-source coupling. Potential residual biases include differences in feature availability after common-space harmonization, mutation/CNV processing, sample ascertainment, treatment history, clinical annotation, and breast-cancer subtype composition. Feature harmonization reduces, but cannot eliminate, these breast-cohort biases; accordingly, the breast results should be interpreted as favorable internal evidence under the available retrospective cohort structure rather than as definitive evidence of population-level generalization. By contrast, the prostate external evaluation was used as an independent-cohort-shift validation test without retraining or additional tuning, and the strong external ranking performance and balanced cross-source behavior support the portability of TF-GateNet in that setting, while still falling short of a prospective population-level estimate.

The interpretability findings should be viewed within the same boundary. Pathway gates, cross-level attribution, and genomic abnormality anchoring provide an inspectable and biologically organized route for hypothesis generation, but they do not by themselves prove causal regulators or validated metastatic mechanisms. The prioritized TF-related cues, genes, and pathways should therefore be regarded as candidate signals that require additional support from independent cohorts, transcriptomic or chromatin-informed measurements, perturbation-based faithfulness tests, and experimental follow-up. This interpretation keeps the biological reading of TF-GateNet aligned with its current evidence level: structured and hypothesis-generating, but not yet causal or experimentally validated.

Future work should therefore focus on three directions. One is multimodal expansion, especially toward transcriptomic or chromatin-informed regulatory measurements that can sharpen the regulatory entry. Another is robustness-oriented evaluation under stronger platform heterogeneity, including targeted panels, cohort rebalancing, and domain adaptation. The third is biological follow-up, in which the recurrent TF-related cues and pathways highlighted by the current interpretability analysis can be prioritized for orthogonal computational or experimental validation. The seed-wise stability analysis added in Supplementary Table S9 addresses training-seed recurrence of prioritized interpretability outputs, but future evaluation would still benefit from perturbation-based faithfulness tests and independent-cohort explanation reproducibility, because these are increasingly regarded as core criteria for judging whether a biology-inspired model is truly interpretable rather than merely biologically readable [11,14].

## 5. Conclusions

TF-GateNet integrates TF-aware feature integration with sample-specific dynamic gating on a hierarchical sparse backbone to predict primary–metastatic state from somatic genomic alterations. Across repeated internal experiments and independent external prostate validation, the model showed its clearest overall advantage on prostate while also delivering the strongest internal ranking performance on the breast cohort. The ablation results further indicate that TF-aware integration accounted for the larger prostate gain, whereas within the TF-GateNet family on breast, both TF-aware integration and dynamic gating contributed positively, with the larger drop observed after removing gating.

More importantly, TF-GateNet was developed not only to improve predictive ranking but also to preserve an inspectable biological decision route. By linking TF-related genomic perturbation cues, genes, pathways, and phenotype-associated predictions within one constrained architecture, the model provides a practical basis for biologically traceable prediction and hypothesis generation. The present evidence therefore supports a focused conclusion: a carefully designed biological structure can improve predictive ranking and interpretation in primary–metastatic state prediction, with the strongest overall generalization evidence observed in prostate cancer and favorable internal evidence also obtained in breast cancer.

## Figures and Tables

**Table 1:** Dataset summary for primary–metastatic state prediction.

Cancer Type	Total	Status Classification		Data Split		
		Primary	Metastasis	Train	Validation	Test
Breast	1572	1111	461	1257	157	158
Prostate	1011	678	333	807	102	102

**Table 2:** Cancer-specific statistics of the analyzed input space and TF regulatory entry used in TF-GateNet.

Cancer Type	Mutation Genes	CNV Genes	Effective TFs	Effective TF–Gene Edges
Prostate	14,378	13,802	448	6769
Breast	4316	19,041	685	13,757

**Table 3:** Main internal test performance across 10 repeated runs on the fixed 80/10/10 split. Values are reported as mean(std). AUROC and AUPRC were treated as the primary metrics, whereas F1, ACC, and MCC summarize threshold-based classification behavior at a fixed metastatic-probability threshold of 0.5. Prostate and breast results are organized as grouped sections within a single table. *q*(AUPRC) denotes the Benjamini–Hochberg-adjusted q-value from paired seed-wise AUPRC comparisons against TF-GateNet within the same cancer cohort.

Model	AUROC	AUPRC	F1	ACC	MCC	*q*(AUPRC)
**Prostate cohort**						
TF-GateNet	**0.954 (0.005)**	**0.925 (0.007)**	**0.825 (0.023)**	**0.886 (0.020)**	**0.747 (0.032)**	Reference
P-NET	0.929 (0.009)	0.875 (0.010)	0.757 (0.041)	0.848 (0.022)	0.664 (0.037)	0.00244
BKGNet-Pathway	0.943 (0.004)	0.893 (0.003)	0.757 (0.041)	0.859 (0.015)	0.678 (0.034)	0.00244
BKGNet-Protein	0.946 (0.002)	0.914 (0.007)	0.799 (0.013)	0.869 (0.011)	0.706 (0.021)	0.00586
FNN	0.927 (0.009)	0.889 (0.017)	0.667 (0.073)	0.826 (0.023)	0.604 (0.051)	0.00244
LR	0.910 (0.000)	0.877 (0.000)	0.691 (0.000)	0.833 (0.000)	0.617 (0.000)	0.00244
RF	0.892 (0.015)	0.814 (0.023)	0.651 (0.033)	0.810 (0.017)	0.555 (0.045)	0.00244
SVM	0.891 (0.000)	0.853 (0.000)	0.696 (0.010)	0.830 (0.005)	0.606 (0.012)	0.00244
XGBoost	0.934 (0.003)	0.912 (0.004)	0.820 (0.005)	0.880 (0.004)	0.731 (0.008)	0.00434
DT	0.851 (0.026)	0.747 (0.024)	0.775 (0.018)	0.846 (0.012)	0.659 (0.027)	0.00244
**Breast cohort**						
TF-GateNet	**0.893 (0.004)**	**0.835 (0.006)**	0.745 (0.044)	**0.856 (0.021)**	**0.652 (0.050)**	Reference
P-NET	0.884 (0.004)	0.819 (0.006)	**0.755 (0.032)**	0.842 (0.035)	0.646 (0.050)	0.00244
BKGNet-Pathway	0.889 (0.005)	0.817 (0.007)	0.747 (0.070)	0.835 (0.102)	0.641 (0.115)	0.00434
BKGNet-Protein	0.883 (0.005)	0.801 (0.008)	0.707 (0.027)	0.834 (0.012)	0.599 (0.025)	0.00244
FNN	0.870 (0.013)	0.794 (0.018)	0.635 (0.080)	0.752 (0.166)	0.502 (0.156)	0.00244
LR	0.866 (0.000)	0.811 (0.000)	0.714 (0.000)	0.848 (0.000)	0.621 (0.000)	0.00244
RF	0.860 (0.015)	0.772 (0.021)	0.600 (0.031)	0.813 (0.013)	0.522 (0.037)	0.00244
SVM	0.858 (0.000)	0.788 (0.000)	0.615 (0.006)	0.817 (0.002)	0.534 (0.006)	0.00244
XGBoost	0.890 (0.004)	0.828 (0.006)	0.750 (0.013)	0.848 (0.006)	0.642 (0.016)	0.02730
DT	0.717 (0.028)	0.561 (0.032)	0.611 (0.029)	0.810 (0.015)	0.515 (0.041)	0.00244

**Table 4:** Ablation of TF-aware feature integration on the prostate and breast cohorts across 10 repeated runs. Values are reported as mean(std); ΔAUPRC and *q*(AUPRC) are computed relative to the full TF-GateNet configuration within the same cancer cohort. *q*(AUPRC) denotes the Benjamini–Hochberg-adjusted q-value from paired seed-wise AUPRC comparisons, and F1 was computed at a fixed metastatic-probability threshold of 0.5.

Cancer Type	Family	TF-Aware Module	AUROC	AUPRC	ΔAUPRC	F1	*q*(AUPRC)
Prostate cancer	P-NET	w/ TF	**0.954 (0.004)**	**0.928 (0.005)**	−0.002	**0.824 (0.031)**	0.5570
	P-NET	w/o TF	0.944 (0.004)	0.898 (0.006)	−0.032	0.771 (0.021)	0.0026
	TF-GateNet	w/ TF	**0.955 (0.005)**	**0.930 (0.007)**	**0.000**	**0.816 (0.024)**	Reference
	TF-GateNet	w/o TF	0.947 (0.004)	0.906 (0.006)	−0.024	0.793 (0.033)	0.0026
Breast cancer	P-NET	w/ TF	**0.888 (0.006)**	0.813 (0.010)	−0.023	**0.730 (0.029)**	0.00391
	P-NET	w/o TF	0.888 (0.004)	**0.832 (0.005)**	**−0.003**	0.728 (0.086)	0.16
	TF-GateNet	w/ TF	**0.893 (0.004)**	**0.835 (0.006)**	**0.000**	0.745 (0.044)	Reference
	TF-GateNet	w/o TF	0.890 (0.004)	0.828 (0.005)	−0.008	**0.747 (0.051)**	0.013

**Table 5:** Ablation of sample-specific dynamic gating on the prostate and breast cohorts across 10 repeated runs. Values are reported as mean(std); ΔAUPRC and *q*(AUPRC) are computed relative to the full TF-GateNet configuration within the same cancer cohort. *q*(AUPRC) denotes the Benjamini–Hochberg-adjusted q-value from paired seed-wise AUPRC comparisons, and F1 was computed at a fixed metastatic-probability threshold of 0.5.

Cancer Type	Family	Dynamic Gating	AUROC	AUPRC	ΔAUPRC	F1	*q*(AUPRC)
Prostate cancer	P-NET	w/ Gate	**0.947 (0.004)**	**0.906 (0.006)**	−0.024	**0.793 (0.033)**	0.0026
	P-NET	w/o Gate	0.944 (0.004)	0.898 (0.006)	−0.032	0.771 (0.021)	0.0026
	TF-GateNet	w/ Gate	**0.955 (0.005)**	**0.930 (0.007)**	**0.000**	0.816 (0.024)	Reference
	TF-GateNet	w/o Gate	0.954 (0.004)	0.928 (0.005)	−0.002	**0.824 (0.031)**	0.5570
Breast cancer	P-NET	w/ Gate	**0.890 (0.004)**	0.828 (0.005)	−0.008	**0.747 (0.051)**	0.013
	P-NET	w/o Gate	0.888 (0.004)	**0.832 (0.005)**	**−0.003**	0.728 (0.086)	0.16
	TF-GateNet	w/ Gate	**0.893 (0.004)**	**0.835 (0.006)**	**0.000**	**0.745 (0.044)**	Reference
	TF-GateNet	w/o Gate	0.888 (0.006)	0.813 (0.010)	−0.023	0.730 (0.029)	0.00391

**Table 6:** External validation on independent prostate cohorts. CR, correct rate; BACC, balanced accuracy; threshold-based metrics were computed at a fixed metastatic-probability threshold of 0.5; values are mean (SD). *q*(AUPRC) denotes the Benjamini–Hochberg-adjusted q-value from paired seed-wise AUPRC comparisons against TF-GateNet.

Model	Primary CR	Metastatic CR	Ext. AUROC	Ext. AUPRC	Ext. ACC	Ext. BACC	*q*(AUPRC)
TF-GateNet	0.891 (0.026)	**0.884 (0.026)**	**0.952 (0.009)**	**0.898 (0.018)**	0.889 (0.018)	**0.888 (0.015)**	Reference
P-NET	0.894 (0.008)	0.868 (0.021)	0.944 (0.004)	0.852 (0.017)	0.888 (0.005)	0.881 (0.009)	0.00195
BKGNet-Pathway	0.913 (0.017)	0.820 (0.056)	0.938 (0.006)	0.830 (0.021)	0.891 (0.010)	0.866 (0.023)	0.00195
BKGNet-Protein	0.834 (0.025)	0.866 (0.040)	0.928 (0.007)	0.830 (0.015)	0.841 (0.013)	0.850 (0.013)	0.00195
FNN	**0.922 (0.027)**	0.835 (0.060)	0.947 (0.012)	0.829 (0.037)	**0.902 (0.016)**	0.878 (0.024)	0.00195
XGBoost	0.799 (0.005)	0.863 (0.006)	0.919 (0.003)	0.831 (0.007)	0.814 (0.005)	0.831 (0.004)	0.00195

## Data Availability

Publicly available datasets analyzed in this study included the curated prostate dataset used in the P-NET study, four cBioPortal-derived breast-cancer cohorts used for internal evaluation, and two cBioPortal-derived prostate cohorts used for external validation. The corresponding cBioPortal study pages are documented in the repository README. The code, selected configuration files, environment specifications, and supplementary result tables used in this study are openly available at https://github.com/Lovin-coder/TF-GateNet (accessed on 30 April 2026) and archived in Zenodo at https://doi.org/10.5281/zenodo.19843641. Supplementary result tables are provided in Supplementary Tables S1–S9. Restrictions apply to the redistribution of part of the source data because these data were obtained from third-party resources and remain subject to the terms of the original providers.

## Supplementary Materials

The following supporting information can be downloaded at: https://www.mdpi.com/article/10.3390/biom16060879/s1, Supplementary Table S1: Complete seed-wise internal benchmarking metrics across the prostate and breast cohorts; Supplementary Table S2: Pairwise statistical comparisons for the main internal benchmarking results across the prostate and breast cohorts; Supplementary Table S3: Complete seed-wise ablation metrics across the prostate and breast cohorts; Supplementary Table S4: Pairwise statistical comparisons for the ablation analyses across the prostate and breast cohorts; Supplementary Table S5: Complete seed-wise external-validation metrics on the independent prostate cohorts; Supplementary Table S6: Pairwise statistical comparisons for the external-validation analyses; Supplementary Table S7: Full ranked interpretability outputs, including TF-related cues, genes, pathways, and risk-bin pathway summaries; Supplementary Table S8: Breast cohort source, retained-sample composition, feature-availability, overlap, final common model-space gene counts, WES/WXS evidence, and potential-bias summary; Supplementary Table S9: Seed-wise interpretability stability summary and recurrent interpretability signals across repeated runs.

## Abbreviations

The following abbreviations are used in this manuscript:

CNV	Copy-number variation
AUROC	Area under the receiver operating characteristic curve
AUPRC	Area under the precision–recall curve
ACC	Accuracy
MCC	Matthews correlation coefficient
BACC	Balanced accuracy
CR	Correct rate
BRCA	Breast-cancer cohort
PRAD	Prostate cancer cohort
LR	Logistic regression
RF	Random forest
SVM	Support vector machine
DT	Decision tree
